# Progress in Cancer

**Published:** 1901-08-24

**Authors:** 


### Progress in Cancer.

At the State Laboratory for the Investigation of Cancer in Buffalo, Gaylord[1] and others have been conducting experiments with a view to discovering the parasite. He gives a paper upon the work already done. He declares that after using fresh methods for some time he has demonstrated certain globular forms which before have been mistaken for fat droplets, but which he finds do not react to potash or ether, nor stain with osmic acid. It also has a different refractive index. These he holds to be the parasites of cancer in one phase of their development. A great number of these organisms can be found in all rapidly-growing tumours. He has produced adenomatous cancer by injecting serum containing these bodies, and set apart in a cool place for as long as four months, into the jugular vein of a guinea-pig. He has great difficulty in recognising them in hardened sections.

Small tumours contain only smaller forms of the organism. Tumours of carcinoma and sarcoma taken from the dead show large numbers of organisms in all stages of development; and on comparing these with tumours removed during life he concludes that either in the period immediately preceding death or after death the organisms proliferate rapidly.

By making successive scrapings of two large-sized tumours, after their removal by operation, he found that the relative size of the organisms increased in size. After 10 hours the amœboid forms were largely increased in number, and after 24 hours spore sacs were present for the first time in great numbers. After observations lasting three days, the sacs appeared to be replaced by hyaline bodies, which were considerably larger than the groups contained by the original sacs.

The "fatty degeneration" of carcinoma may therefore be in part due to the organisms mistaken for fat droplets; and epithelial cells thought to be in a state of advanced fatty degeneration may be occupied by these parasites. The so-called "cancer milk" is practically a pure culture of these organisms. He has incubated a hanging drop on a warm stage, and has found the forms change the one into the other, the smaller round forms changing to the larger forms, which become granulated, throw out pseudopodia and develop a nucleus (amœboid form) and end by turning into a sac containing spores.

On using fixatives nearly all forms disappear except the smaller ones, recognised first by Russell and called "Fuchsin bodies," the larger forms when stained closely resembling free nuclei, or round cells, and so are difficult to distinguish. He found in a case of peritonitis and cancer of the uterus that the peritoneum, fluids, tissues, and blood were bacteriologically free, but fresh preparations were full of these organisms. Since this he has found all organs, and blood from all regions, of all cases dead of sarcoma or carcinoma contain these organisms.

Where cachexia is marked living cases show the younger forms in the peripheral blood, showing well oscillating motion; while the quarter-grown organisms are actively sending out pseudopodia, and resemble closely those organisms found in blood by Pfeiffer and Reed after vaccination and smallpox. Animals can be inoculated with this blood.

Gaylords give a *résumé* of the recent [discoveries by Sanfelice, Plimmer, and others, and endorses their discoveries, while he gives reasons for considering that Plimmer's bodies cannot be cell degenerations. He traces a direct morphological relation between Plimmer's and Russell's bodies. He has failed to find blastomycetes; thus far yeasts, in his hands, have shown mostly a double contour unlike the Plimmer organism, and he states that "From a morphological standpoint he has been unable to identify Plimmer's bodies with transformed yeast organisms."

The half-grown form of vaccine organism is very closely related in appearance to Plimmer's and Russell's bodies; and the protozoon forms in carcinoma have prototypes in various stages of development in the vaccine organism. Gaylord has little doubt that the organism is a protozoon. He, however, awaits evidence that this organism which can be found constantly in all forms of cancer is the actual cause of the disease. He is about to publish the results of numerous inoculation experiments, and has already shown that the virulence of the organism is increased by its passage through guinea-pigs. Thus he has shown that these animals when inoculated from human cancer die, on an average, in 45 to 58 days, while those inoculated from the infected guinea-pigs themselves die in 29 days. Rabbits show greater resistance. The parasites may be recovered from their blood and organs. Animals may be inoculated as well from dry lymph nodes as with recent material.

According to Roswell Park[2] the cancer parasite can be found in the regional lymph nodes, before the epithelial cells themselves can be detected. He states that animals inoculated with sarcoma, are not devoid of epithelial lesions, and *vice versâ*. Cutten has pointed out that myoma and uterine cancer are associated, though clinically this is rare. Park draws attention to the "terminal infection" of cancer, death being caused by a distinct toxæmia or hæmatogenous infection. As the malarial parasite is inoculated by the mosquito, Nason[3] suggests that some other blood-sucking insect may be accountable for transmitting cancer in certain cases.

Different forms of parasites in sarcoma and carcinoma are also described by Professor Max Schüller.[4] A young small golden-coloured form, and a larger which consists of a capsule enclosing smaller forms, the one probably developing into the others. Borrel,[5] on the other hand, has found that a phase in the development of the guinea-pig's sporozoon reveals a condition identical in appearance with the cell-inclusious of cancer. He considers that the yeasts isolated are contaminations, and that there is a tendency "to range everything that is round and contains a capsule among the blastomycetes."

Tumour formation according to Brosch[6] is the outcome of a destructive influence reacting upon a tissue in which the productive process already in progress is more than that required for tissue repair, such as after trauma. He suggests that deep wounds are followed by sarcoma, superficial by carcinoma. Following upon Behla's remarkable article upon the distribution of cancer cases in the township of Luckau, Lyon,[7] of Buffalo, has investigated the prevalence in the city of Buffalo during the 20-year period 1880–1899. Lyon points out that the incidence of cancer in that city is greater among the foreigners living in its precincts than among the natives. The largest death rate is among the Germans, while the Italians are proportionately free. This is not due, he says, to the density of populous districts, as the Italian quarter is more densely populated than the German parts. Unlike Behla, he finds no watercourses, ditches, etc., to account for this, but he finds that the Germans eat largely of raw vegetables, as those in Luckau; and this may find support as a cause in the fact that the Germans are more prone to cancer of the stomach and liver, while that of the uterus, etc., does not show a disproportionate increase. He is of opinion that race *per se* has much to do with the incidence. He finds that the increase rate is 32–53 per 100,000 inhabitants. He concludes that these statistics support the parasitic as opposed to the embryonic view of infection.

Bryant[8] quotes statistics from asylums to show that extreme mental depression cannot be thereby regarded as a proved ætiological factor in malignant disease. Cullen[9] states that the presence of large coal-black cells in the curette scrapings of the uterus is pathognomic of cancer.

In *American Medicine* Soler[10] emphasises the importance of the secondary manifestations of cancer in the cerebro-spinal region. Sometimes the original tumour is overlooked, and then the spinal symptoms may be puzzling. Herpes zoster may be present, with painful symptons and paralysis. In a case of his own after complete paralysis, sensory and motor, power of walking returned, through possibly the absorption of the secondary growth.

Variation in size, and variation in tenderness are given by Marmaduke Shield[11] as the two important points in the diagnosis of cyst from cancer of the breast. Axillary glands are deceptive: they may be too deeply buried in fat to be easily felt, or they may be enlarged from other causes than cancerous infection (iodine, etc.).

According to Coley[12] himself, spindle-celled sarcomata react best to the "mixed toxins." If there is no response to treatment after three or four weeks then there is no use in continuing. There is practically no risk; three to four per cent. round-celled sarcomata are cured, and 3·0 per cent. of the spindle-celled variety. It is of little avail in the melanotic and lymphoid sarcomata, or in carcinoma. Cacadylate of soda is recommended by Paque[13] in inoperable cancers. He suggests that the action of arsenic, may be at first stimulating and eventually destructive to epithelial tissues. Hence in large doses it may be detrimental to cancer. He has given up to 7·5 centigrammes hypodermically. Jonathan Hutchinson[16] speaks of arsenic as being the only remedy to arrest the progress of multiple sarcomatous tumour (Hodgkin's disease). He gives 6–8 m. liquor fowleri three times daily until it disagrees. Andrew Clark[14] records a case where material improvement seemed to follow the use of X-rays in a case of inoperable recurrent cancer, while Williams[15] of Boston has treated several cases of cutaneous cancer with the rays successfully. He warns against producing burn, and states that some time is required to effect a cure.

[1] Amer. Jour. Med. Sci., May, 1901; Brit. Med. Jour., April 27; Lancet, May 18. [2] Med. Rec., May 18. [3] Brit. Med. Jour., May 18. [4] Brit. Med. Jour., May 25. [5] Brit. Med. Jour., June 8. [6] Brit. Med. Jour., June 1. [7] Amer. Jour. Med. Sci., June, 1901. [8] Med. Rec., May 18. [9] Med. Rec., May 18. [10] Lancet, May 25. [11] Brit. Med. Jour., May 18. [12] Med. Rec, May 18. [13] Lancet, May 25. [14] Brit. Med. Jour., June 8. [15] Brit. Med. Jour., June 8. [16] Olin. Jour., May 22.

